# Maternal and neonatal omentin-1 levels in gestational diabetes

**DOI:** 10.1007/s00404-018-4652-5

**Published:** 2018-01-15

**Authors:** Marie Franz, Mariella Polterauer, Stephanie Springer, Lorenz Kuessel, Peter Haslinger, Christof Worda, Katharina Worda

**Affiliations:** Department of Obstetrics and Gynecology, Medical University of Vienna, Währinger Gürtel 18-20, 1090 Vienna, Austria

**Keywords:** Omentin-1, Gestational diabetes, Adipokines, Insulin resistance

## Abstract

**Purpose:**

To evaluate the effect of gestational diabetes on omentin-1 in maternal and cord plasma. As a potent mediator of insulin resistance, Omentin-1, an adipokine derived from human adipose and placental tissue, may be an important player in the pathophysiology of gestational diabetes.

**Methods:**

This was a prospective case–control study. The study included 96 women with gestational diabetes and 96 pregnant women without. Omentin-1 was measured at the time of the oral glucose tolerance test, at 32 weeks in maternal plasma and right after delivery in umbilical cord blood by ELISA assay.

**Results:**

Over a period of 2 years, 200 patients were enrolled. Omentin-1 levels did not significantly differ between both groups throughout the pregnancy: omentin-1 levels were 157 ± 83 ng/ml in women with gestational diabetes and 158 ± 93 ng/ml in women without gestational diabetes (*p* = 0.94) at time of the oral glucose tolerance test and 118 ± 77 ng/ml in women with diabetes and 150 ± 89 ng/ml in women without (*p* = 0.12) at 32 weeks, respectively. Both groups showed a decrease in omentin-1 levels throughout pregnancy, with a more pronounced decrease in diabetic women (13 ± 53 versus 4 ± 48 ng/ml; *p* = 0.5). Neonatal omentin-1 levels were significantly lower in offspring of diabetic mothers: 106 ± 61 versus 134 ± 45 ng/ml (*p* = 0.03).

**Conclusions:**

There was no significant difference in omentin-1 levels between healthy and diabetic mothers throughout the pregnancy. However, we found significantly lower omentin-1 levels in offspring of diabetic mothers. This may indicate a risk for the development of insulin resistance in later life.

## Introduction

In each pregnancy, a physiological insulin resistance syndrome occurs to ensure that the fetus is sufficiently supplied with glucose. Despite this physiological insulin resistance, most women stay normoglycemic throughout the pregnancy because of adequate β-cell function. If this physiological compensation fails, gestational diabetes occurs.

Gestational diabetes is one of the most common pregnancy-associated diseases with a prevalence of 5–10% of all pregnancies and its prevalence is increasing [1, 2]. It includes facets of type 2 diabetes (DM2) like insulin resistance and up to 50% of women with GDM develop DM2 within the following 10 years after pregnancy [3, 4]. Gestational diabetes is associated with severe hazards to both mother and fetus such as macrosomia, plexus palsy, premature delivery, and intrauterine death [5, 6]. Furthermore, the exact pathogenesis of GDM is not completely understood; however, increased insulin resistance is a well-demonstrated mechanism [7].

A recent study suggests that not only hyperglycemia but also altered maternal lipid metabolism may constitute a risk factor for macrosomia in GDM [8].

It has been described that adipokines, which are proteohormones secreted mainly from adipose tissue, could play an important role in the development of diabetes and also gestational diabetes [9]. Adipokines influence metabolic processes through various pathways like appetite control, inflammation, regulation of adipogenesis, and alter insulin sensitivity and secretion [9]. Since several adipokines like leptin and adiponectin are already investigated quite well, data about omentin-1 in gestational diabetes are rare and conflicting.

Omentin-1 is derived from human adipose tissue as well as placental tissue [1] and has been shown to be a potential mediator of insulin resistance. It was first described by Yang et al. as a visceral-fat specific secretory factor. In detail, they demonstrated that treatment with recombinant omentin-1 raised insulin-stimulated glucose transport in vitro, suggesting that omentin-1 may improve insulin sensitivity [10].

Later on, omentin-1 was shown to be downregulated by insulin and glucose, resulting in decreased levels in overweight women suffering from polycystic ovary syndrome [11]. Furthermore, decreased omentin-1 levels were found in patients suffering from obesity and diabetes [12].

This study was conducted to illuminate a possible association between the adipokine omentin-1, gestational diabetes, and lipid metabolism.

## Materials and methods

The Ethics committee of the Medical University of Vienna approved the study protocol. Informed consent was obtained from all individual participants included in the study before recruitment. The study was performed according to the standards of the Helsinki Declaration of 1964 and its later amendments. This study is registered with clinicalTrials.gov, http://www.clinicaltrials.gov, NR 11072.

**Fig. 1:**
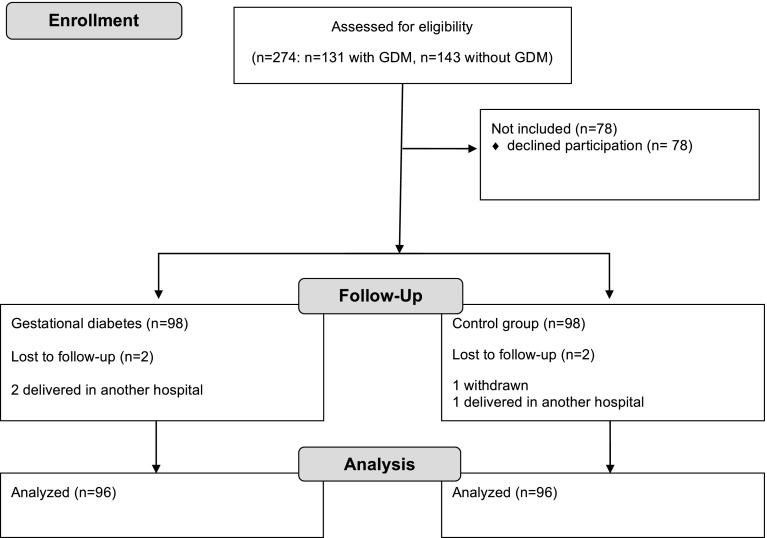
Screening, enrollment, and follow-up of the study participants

Over a period of 2 years, 276 pregnant women were asked to participate in the study; of those 200 agreed. A flow chart of the selection process is shown in Fig. 1. Sample size of 90 persons per group was calculated with a power of 0.90 and a type one error of 0.05 with mean differences between the groups of 5 ng/ml and a standard deviation of 10.

Ninety-six pregnant women with diagnosed gestational diabetes mellitus (Study group) and ninety-six pregnant women with normal oral glucose tolerance test (OGTT) were consecutively enrolled into the study. Recruitment was performed at the outpatient clinic of the Department of Obstetrics and Gynaecology at the University Hospital of Vienna, which is a tertiary care center serving high-risk pregnancies with different pregnancy-associated complications. Patients with chronic diseases, pre-existing diabetes mellitus or hypertension, as well as multiple pregnancies or pregnancies complicated by fetal anomalies were being excluded from the study.

A standard oral glucose tolerance test (OGTT) with 75 g glucose according to the criteria of the American Diabetes Association was performed between 24 and 28 weeks of pregnancy in all patients. The new guidelines of the German and the Austrian Society for Diabetes (modified Carpenter Coustan Criteria) adjusted for the results of the HAPO study [13] have been implemented for the evaluation of the OGTT. Upper normal limits for fasting glucose, 1 and 2 h after glucose ingestion, were 92, 180, and 153 mg/dl, respectively. Prior to the test, a precise medical and obstetric history, including patients’ body mass index (BMI), was assessed in a structured personal interview.

Omentin-1 values were measured at two time points during pregnancy (at the time of the OGTT and at 32 weeks) and in umbilical cord blood right after delivery. Maternal blood sampling included a 10 ml plasma sample which was spinned and stored at − 80 °C until the final assessment of omentin-1 levels. Furthermore, maternal lipid levels (total cholesterol, low-density lipoprotein cholesterol, high-density lipoprotein cholesterol, and triglycerides) were assessed. If gestational diabetes was diagnosed, a patient routine treatment (dietary recommendations and insulin therapy if needed) was initiated. In our routine care, patients visit the outpatient department approximately every third week to obtain a routine ultrasound for fetal weight estimation and check-up of the recorded blood glucose levels. In case of insulin-dependent gestational diabetes, labor is induced at term.

Cord blood samples were collected right after delivery from the placental part of the umbilical cord and immediately centrifuged after collection and stored at − 80 °C until assay.

The determination of omentin-1 levels was performed by Enzyme-linked Immunosorbent Assay Kit (ELISA Uscn Life Science Inc., Wuhan, China).

### Statistical analysis

Parametric continuous variables are summarized as means (± standard deviation), non-parametric continuous variables as medians (minimum and maximum) and categorical data as percentages.

Comparison of groups was done by Kruskal–Wallis test and pairwise comparison was performed by Mann–Whitney *U* test. Chi-square test was used for comparisons of proportions. Logistic and linear regression analyses were used appropriately. All tests were two-tailed and an alpha-level of *p* < 0.05 was considered statistically significant. All statistical analyses were being performed using the statistical software package SPSS 18.0 (SPSS Inc., Chicago, IL, USA).

## Results

The demographic data of mothers and infants are shown in Tables 1 and 2. Women with GDM had a higher BMI and delivered earlier than women with normal glucose tolerance. They did not significantly differ from women without GDM in maternal age, HbA1c levels, and lipids. Linear regression analysis showed that BMI was an independent risk factor for GDM (*p* = 0.006). Offspring of mothers with GDM had higher serum C-peptide levels than offspring of mothers without GDM. Birth weight did not significantly differ between both groups.

**Table 1:** Patients’ characteristics

	GDM (*n* = 96)	nGDM (*n* = 96)	*p* value
Age (years)	34.1 ± 7.4	32.8 ± 9.3	0.16
BMI (kg/m^2^)	28.0 ± 6.6	26.3 ± 4.7	0.01
Blood glucose level fasting (mg/dl)	94 ± 13	77 ± 9	< 0.001
1 h postload glucose (mg/dl)	178 ± 31	126 ± 29	< 0.001
2 h postload glucose (mg/dl)	139 ± 33	102 ± 24	< 0.001
GA visit 1 (wop)	26.4 ± 1.3	26.3 ± 1.4	0.86
GA visit 2 (wop)	32.7 ± 0.9	32.4 ± 0.9	0.10
GA at delivery (wop)	38.5 ± 1.9	39.1 ± 2.0	0.01
Maternal HbA1c at visit 1	5.43 ± 0.84	5.42 ± 0.43	0.94
Maternal HbA1c at visit 2	5.44 ± 0.41	5.27 ± 0.45	0.29
TG at visit 1	298 ± 133	271 ± 112	0.32
HDL at visit 1	65 ± 12	69 ± 15	0.12
LDL at visit 1	154 ± 34	156 ± 65	0.93
Total cholesterol at visit 1	269 ± 55	267 ± 62	0.96

*BMI* body mass index, *GA* gestational age, *wop* weeks of pregnancy, *TG* triglycerides, *HDL* high-density lipoprotein cholesterol, *LDL* low-density lipoprotein cholesterol

**Table 2:** Neonatal characteristics

	GDM	nGDM	*p* value
5 min APGAR	9.6 ± 0.8	9.8 ± 0.5	0.18
pH	7.26 ± 0.08	7.24 ± 0.12	0.20
Birthweight (g)	3240 ± 460	3215 ± 574	0.80
C-peptide	3.6 ± 1.57	2.06 ± 1.03	0.04

*GDM* gestational diabetes, *nGDM* no gestational diabetes

Omentin-1 levels are shown in Table 3. In summary, omentin-1 levels did not significantly differ between both groups throughout the pregnancy: omentin-1 levels were 157 ± 83 ng/ml in women with GDM and 158 ± 93 ng/ml in women without GDM (*p* = 0.94) at time of the oGTT and 118 ± 77 ng/ml in women with GDM and 150 ± 89 ng/ml in women without GDM (*p* = 0.12) at 32 weeks, respectively. Both groups showed a decrease in omentin-1 levels throughout pregnancy, with a more pronounced decrease in women with GDM (13 ± 53 versus 4 ± 48 ng/ml; *p* = 0.5).

**Table 3:** Omentin-1 levels

	GDM	nGDM	*p* value
Omentin-1 levels at 26 wop (ng/ml)	157 ± 83	158 ± 93	0.94
Omentin-1 levels at 32 wop (ng/ml)	118 ± 77	150 ± 89	0.12
Omentin-1 levels in cord blood (ng/ml)	106 ± 61	134 ± 45	0.03

*GDM* gestational diabetes, *nGDM* no gestational diabetes, *wop* weeks of pregnancy

Linear regression analyses including maternal age, BMI, HbA1c, low-density lipoprotein cholesterol (LDL), high-density lipoprotein cholesterol (HDL), triglycerides, and total cholesterol showed that BMI and HDL levels significantly influenced omentin-1 levels at 33 weeks. Women with higher BMI and lower HDL had lower omentin-1 levels at 33 weeks (*p* = 0.04; *β* = − 0.18 and *p* = 0.02; *β =* 0.25, respectively). All other parameters were discarded by the regression model.

Fetal omentin-1 levels measured in cord blood after delivery were significantly lower in offspring of diabetic mothers compared to those without diabetes: 106 ± 61 versus 134 ± 45 ng/ml (*p* = 0.03). Fetal omentin-1 levels did not correlate with birth weight and mode of delivery (Pearson’s correlation coefficients 0.08; *p* = 0.39 and 0.15; *p* = 0.11, respectively).

## Discussion

Although the study was adequately powered, maternal lipids and omentin-1 levels did not differ between women with and without GDM. In both groups, omentin-1 levels decreased throughout pregnancy. We have shown that women with higher BMI have lower omentin-1 levels. Offspring of diabetic women had significantly lower omentin-1 levels than those of mothers without diabetes.

It has been reported that omentin-1 levels decrease with the increase of visceral obesity, which is in accordance to our results showing lower omentin-1 levels in obese women [14, 15].

In the literature, there are two studies on omentin-1 and its potential role in gestational diabetes: in one cohort, similar omentin-1 concentrations between patients suffering from GDM and controls have been observed [16], whereas one cohort showed decreased concentrations in GDM [17].

In accordance to Barker et al., we showed lower omentin-1 levels in obese women, but not in women with GDM [16]. Furthermore, we showed a decrease in omentin-1 levels throughout pregnancy. That seems unexpected regarding the fact that the placenta additionally produces omentin-1 [16]. An increased clearance in the later stage of pregnancy or physiological hemodilution during gestation could be an explanation for the decrease of omentin-1 levels throughout pregnancy, which needs further evaluation. On the other hand, it has been shown that omentin-1 is downregulated by insulin and glucose [14]. Therefore, one could assume that the changed insulin sensitivity during pregnancy could lead to decreased omentin-1 levels in the third trimester in both—normoglycemic women as well as women with GDM.

One of the main short-term complications in offspring of diabetic mothers is the higher rate of macrosomia [13]. In our cohort, birth weight did not significantly differ between both groups, probably explained by significantly lower gestational age at birth in the diabetic group.

Regarding long-term complications, it has been reported that offspring of mothers with GDM have greater risk of obesity, the metabolic syndrome, and type 2 diabetes in later life [18–20]. We found significantly lower omentin-1 levels and higher serum C-peptide levels in offspring of mothers with GDM. This could reflect an already altered metabolic state possibly associated with a greater risk of adverse metabolic sequelae in childhood and adult life. This is supported by the study of Catli et al., demonstrating that omentin-1 is significantly lower in obese children compared to normal-weight ones [21]. On the contrary, another study investigating obese children and adolescents did not show altered omentin-1 levels [22].

In conclusion, the clear role of maternal omentin-1 levels in the pathophysiology of gestational diabetes remains to be elucidated.

However, our results regarding decreased omentin-1 levels in offspring of diabetic mothers may indicate a risk for the development of insulin resistance in later life.
